# Development of an automated radiotherapy dose accumulation workflow for locally advanced high‐risk prostate cancer – A technical report

**DOI:** 10.1002/jmrs.442

**Published:** 2020-10-15

**Authors:** Ashley Ong, Kellie Knight, Vanessa Panettieri, Matthew Dimmock, Jeffrey Kit Loong Tuan, Hong Qi Tan, Zubin Master, Caroline Wright

**Affiliations:** ^1^ Department of Radiation Therapy National Cancer Centre Singapore; ^2^ Department of Medical Imaging and Radiation Sciences Monash University Clayton Australia; ^3^ Alfred Health Radiation Oncology Alfred Hospital Melbourne Australia

**Keywords:** Deformable image registration, dose accumulation, prostate cancer, whole‐pelvis radiotherapy, workflow

## Abstract

An automated dose accumulation and contour propagation workflow using daily cone beam computed tomography (CBCTs) images for prostate cases that require pelvic lymph nodes irradiation (PLNs) was developed. This workflow was constructed using MIM® software with the intention to provide accurate dose transformations for plans with two different isocentres, whereby two sequential treatment phases were prescribed. The pre‐processing steps for data extractions from treatment plans, CBCTs, determination of couch shift information and management of missing CBCTs are described. To ensure that the imported translational couch shifts were in the correct orientation and readable in MIM, phantom commissioning was performed. For dose transformation, rigid registration with corrected setup shifts and scaled fractional dose was performed for pCT to daily CBCTs, which were then deformed onto CBCT_1_. Fractional dose summation resulted in the final accumulated dose for the patient allowing differences in dosimetry between the planned and accumulated dose to be analysed. Contour propagations of the prostate, bladder and rectum were performed within the same workflow. Transformed contours were then deformed onto daily CBCTs to generate trending reports for analysis, including Dice Similarity Coefficient (DSC) and Mean Distance to Agreement (MDA). Results obtained from phantom commissioning (DSC = 0.96, MDA = 0.89 mm) and geometrical analysis of the propagated contours for twenty patients; prostate (DSC: 0.9 ± 0.0, MDA: 1.0 ± 0.3 mm), rectum (DSC: 0.8 ± 0.1, mm, MDA: 1.7 ± 0.6 mm) and bladder (DSC: 0.8 ± 0.1, MDA: 2.8 ± 1.0 mm) were within clinically accepted tolerances for both DSC (>0.8) and MDA (< 0.3 mm). The developed workflow is being performed on a larger patient cohort for predictive model building, with the goal of correlating observed toxicity with the actual accumulated dose received by the patient.

## Graphical abstract

An automated dose accumulation and contour propagation workflow using daily cone beam computed tomography images for prostate cancer was developed.

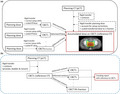

## Introduction

The management of high‐risk prostate cancer (HR‐PCa) typically involves a course of radiotherapy with a total dose prescription of 74–78 Gy in addition to hormonal therapy treatment.^1^ The majority of the patients will have only one treatment plan generated on the planning CT (pCT) data set prior to treatment. As such, daily variations in organ deformations throughout the course of radiotherapy are not taken into account.^2^ The dose received by the prostate and organs at risk (OARs) might be either over or under‐estimated, which has the potential to impact on the local control and radiation‐induced toxicity of the patient.^3^

In recent years, there has been growing interest in using deformable image registration (DIR) for dose accumulation and contour propagation to obtain a more accurate dose estimate of the OARs and target.^3^ To the best of our knowledge, the development of an automated dose accumulation and contour propagation workflow for PCa that involves two treatment phases has not been reported. The purpose of developing the workflow is to facilitate the generation of accurate accumulated dosimetric data for the construction of a predictive model that correlates with the occurrence of toxicities on a larger cohort of HR‐PCa patients. The focus of this technical report is to describe the development of a workflow using a commercial software package for PCa with a two‐phased treatment regimen. The pre‐processing steps for data extractions from treatment plans, CBCTs, determination of couch shift information and management of missing CBCTs are described.

## Methods

This workflow was developed for a retrospective study to analyse planned versus delivered dose for patients with HR‐PCa treated between 2016 and 2018. The study was approved by SingHealth Centralised Institutional Review Board (CIRB Ref: 2019/2018), Singapore.

### Deformable image registration (DIR) algorithm

MIM v.6.9 (MIMVista Corp., Cleveland OH) was used to develop the workflow. MIM DIR is an intensity‐based free‐form deformable algorithm that utilises the sum of squared difference between voxel HU values (per voxel), which seeks to minimise intensity differences between two images for image registration.^4^, ^6^ This was a sequential treatment regimen whereby a dose of 46–54 Gy in 23‐27 fractions was prescribed to the prostate, seminal vesicles (SVs) and pelvic lymph nodes (PLNs) in Phase 1 (P1). A cone down Phase 2 (P2) dose of 24–28 Gy in 12–14 fractions was given to the involved SVs and prostate.

### Pre‐processing steps

**Figure 1:**
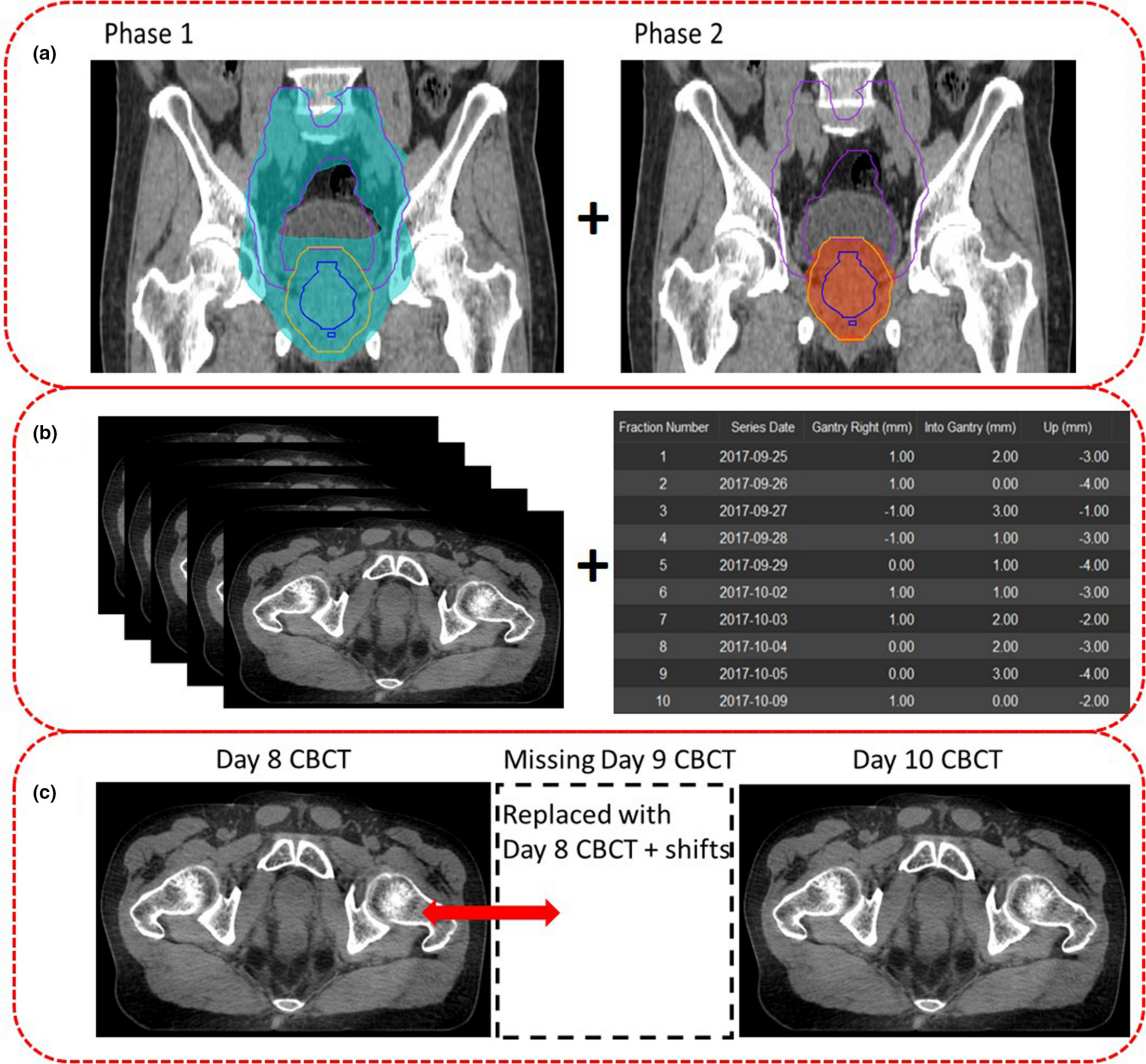
Pre‐processing steps prior to performing dose accumulation. (A) RT plan information from Eclipse TPS, (B) Daily CBCT images and treatment couch shifts information, (C) CBCT data management.

A customised workflow was created in MIM to perform both dose accumulation and contour propagations for PCa using daily CBCTs. The pre‐processing steps are illustrated in Figure 1.

#### Data extraction – Treatment plans and CBCTs

Treatment plan information (RTplan, RTdose and RTstructures) for P1 and P2 were exported from the Eclipse Treatment Planning System^®^ (TPS) (Varian Medical Systems) and imported into MIM. In the electronic medical record (Mosaiq^®^, Elekta) interface, scripting was used to extract patients' daily CBCT images with updated study descriptions to indicate P1/P2 and associated fraction number for easy identification (Fig. 1A and B).

#### Treatment couch shifts extraction

Scripting was used to auto‐populate the couch shift information, CBCT unique identifiers (UID) and CBCTs series dates in comma‐separated values (.csv) format from the Mosaiq server as recommended by the MIM engineers (Fig. 1B).

#### CBCT data management

To ensure that the total number of CBCTs equated to the prescribed number of treatment fractions, any missing CBCTs on a particular day were replaced by the previous day CBCT with the associated shift information; it was assumed that organ deformation remained similar to the closest temporal match (Fig. 1C). Five patients had one missing CBCT each. This accounted for 5 (2.7%) out of 185 CBCTs being replaced.

### Couch shifts commissioning

**Figure 2:**
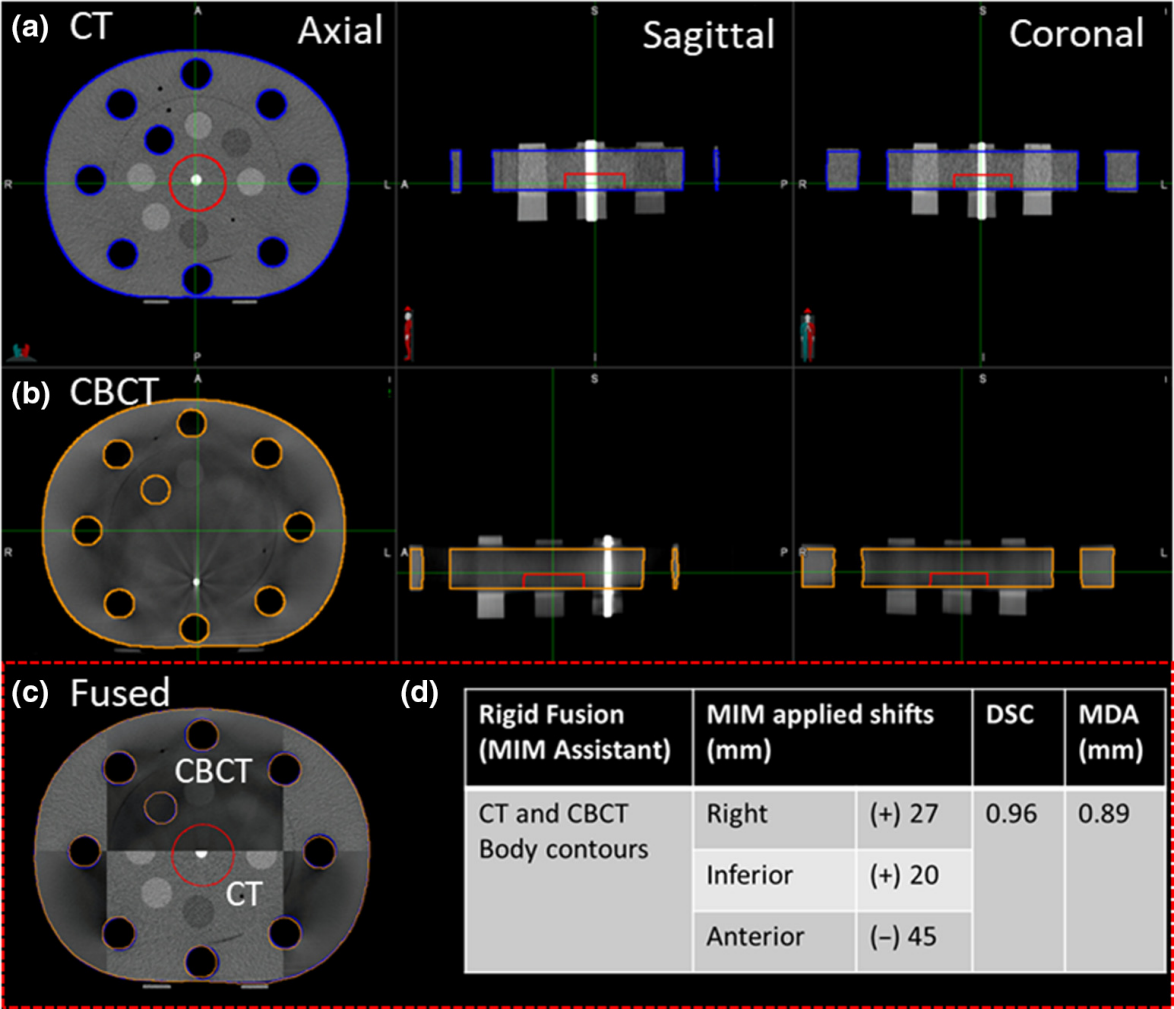
(A) CT body contour (Blue) and (B) CBCT body contour (orange) in three planes. (C) Checker‐box displaying the overlay between CT and CBCT images. (D) Rigid fusion results measured using DSC and MDA.

To ensure that the extracted shifts from Mosaiq coincided with the shift orientations based on the International Electrotechnical Commission (IEC) fixed coordinate system used in MIM, couch shift commissioning was performed as follows:

1. A CIRS model 62 phantom (CIRS Tissue Simulation Technology, Norfolk, VA, USA) was first scanned on a CT‐simulation scanner.
2. CT images were imported into the Eclipse TPS whereby a target volume was contoured at the centre of the phantom. A single anterior plan with 6 MV energy and a field size set at 10 × 10 cm^2^ and isocentre positioned at the centre of the target was generated. A quality assurance (QA) phantom ‘patient’ was created in Mosaiq for plan export and delivery at the treatment unit using a Trilogy linear accelerator (Varian Medical Systems, Palo Alto, CA).
3. Prior to CBCT acquisition, large translational shifts (≥ 2 cm) were performed. Rigid registration was undertaken between the pCT and the acquired CBCT images using the auto‐match tool at the four‐dimensional integrated treatment console (4DITC).
4. Translational shifts were applied and transferred into Mosaiq. Shift information in csv files and the acquired phantom CBCT were extracted from Mosaiq and imported into MIM.
5. Body contours of the pCT and CBCT were segmented (Fig. 2A and B).
6. For couch shift commissioning, a rigid registration was performed between the pCT and CBCT by keying in the shifted couch values obtained at the treatment unit in the shift editor.
7. As per our departmental image‐guided radiotherapy (IGRT) pelvis protocol, only translational shifts were applied. For P1, daily online shifts were applied based on the prostate only shifts, if the bony match shifts were less 5 mm prior to delivery. Patients were re‐aligned/setup again if prostate to bony match exceeded 5 mm (due to patient's movements). For P2, matching was undertaken based on prostate position.
8. Geometrical metrics Dice Similarity Coefficient (DSC) and Mean Distance to Agreement (MDA) were used to measure the fusion accuracy between pCT and CBCT (Fig. 2D).

### Creating a customised workflow

A customised workflow using the command script was first created in the workflow builder for dose transformation and contour propagation within MIM.

#### Dose transformation

**Figure 3:**
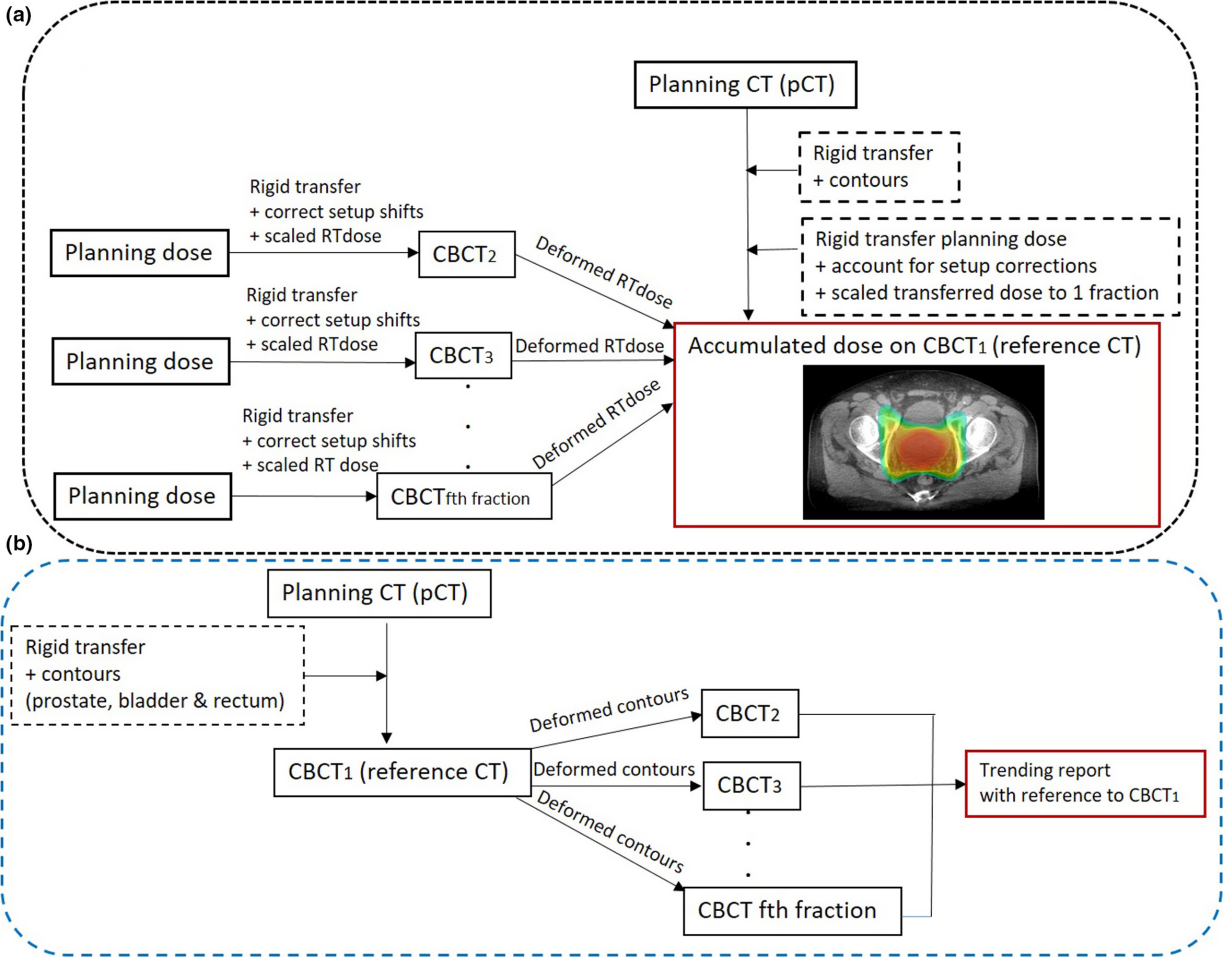
(A) Dose accumulation process using the pCT and daily CBCTs. (B) Contour propagation process within the workflow.

The pCT dose was rigidly transformed onto daily CBCTs and subsequently scaled to a fractional dose of 2 Gy after accounting for treatment setup corrections (Fig. 3A). The fractional dose on each CBCT was then deformed onto the patient's reference geometry (CBCT_1_) volume so that deformable dose accumulation could be performed.

#### Contour propagation

Within the dose accumulation workflow, contour propagations of the prostate, rectum and bladder were performed using a rigid transformation of contours from the pCT to CBCT_1_, prior to deforming onto the subsequent incoming data set CBCT_f_ where f = {2,3,…F} and F is the total number of fractions (Fig. 3B). Based on clinical experience, rigid transfer of contours from pCT to CBCT_1_ was performed as minimal variations in bladder and rectum volumes were expected due to the short period (average 9 days) from CT scan to first day of treatment. Additionally, patients were educated to adhere to rectum emptying and bladder filling protocol.^5^ Geometrical analysis (DSC and MDA) was performed on MIM propagated contours with radiation oncologist (RO) delineated contours as baseline. This workflow was conducted within MIM Assistant's interface whereby an environment for automation of data transfer and workflow management operates by creating rules and filters.^6^

## Results

The workflow was tested successfully on 20 HR‐PCa prostate cases. The results for one case will be further discussed in this technical report to demonstrate the practical application of this workflow. Geometrical analysis of the contours for 20 cases will also be presented.

### Shift commissioning

The achieved DSC (0.96) and MDA (0.89 mm) for accuracy metrics measurements as described in section 2.3 using phantom validation were within clinically accepted tolerances (DSC> 0.8, MDA < 3 mm).^7^

### Dose accumulation

**Figure 4:**
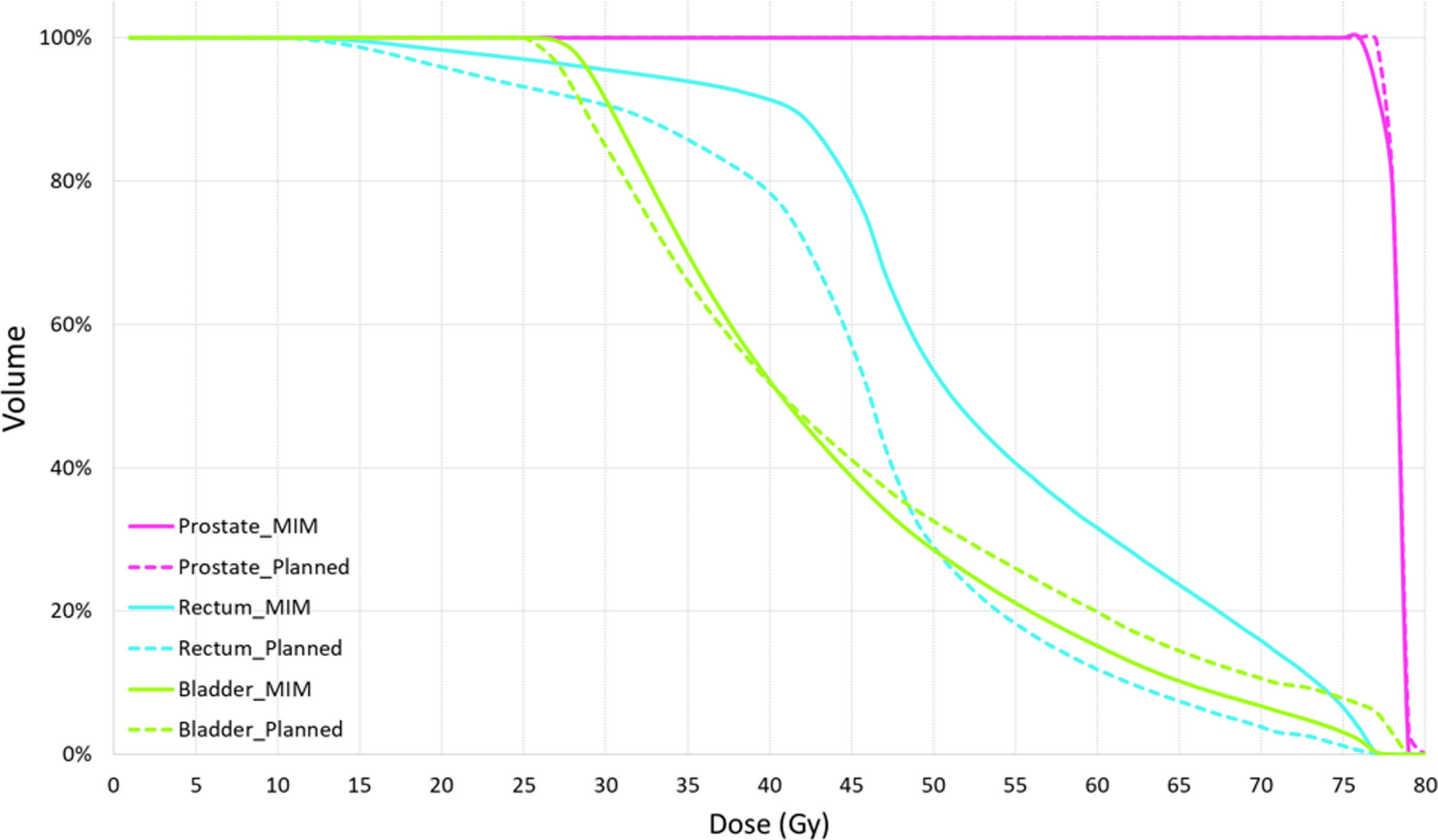
Accumulated dose generated by MIM (solid line) and pCT dose (dashed line) for one example prostate case.

The developed workflow was able to extract final accumulated dose data of the prostate and OARs for analysis (Table 1). Dose volume histograms (DVHs) were generated to demonstrate the dose difference between the MIM accumulated dose and the planned dose (Fig. 4).

**Table 1:** Results obtained from MIM accumulated dose and planned dose for one example prostate case.

Parameters	MIM Prostate	Planned Prostate	MIM Bladder	Planned Bladder	MIM Rectum	Planned Rectum
D98% (Gy)	75.8	75.4				
D95% (Gy)	75.1	75.6				
D_min_ (Gy)	75.3	74.6				
D_mean_ (Gy)	76.4	76.4				
V75Gy (%)			2.0	6.0	3.6	0.1
V70Gy (%)			6.0	9.7	14.2	2.8
V65Gy (%)			9.4	13.6	22.1	6.6
V60Gy (%)			14.0	18.6	30.0	10.9
V55Gy (%)			19.8	24.7	38.7	16.7
V50Gy (%)			27.0	31.2	50.3	26.2
D_mean_ (Gy)			43.4	44.2	52.8	45.0

Abbreviations: D_min_(Gy): minimum dose received by a structure and D_mean_(Gy); Dx%(Gy): dose delivered to x volume of a structure; mean dose received by a structure; VxGy(%): volume of structure receiving a dose of xGy.

### Contour propagation

**Figure 5:**
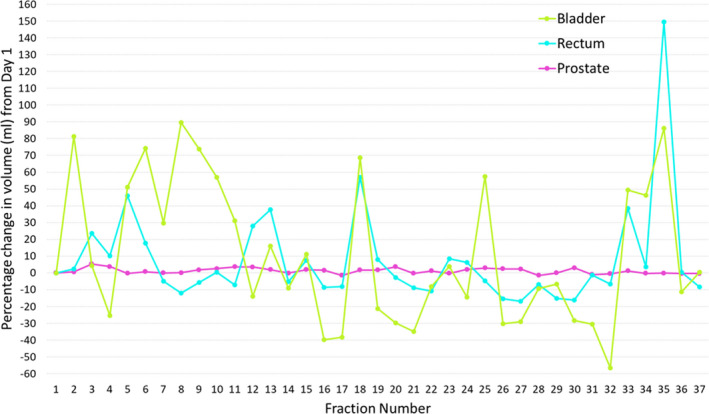
Trend analysis of the change in prostate and OAR volumes for one example prostate case.

**Figure 6:**
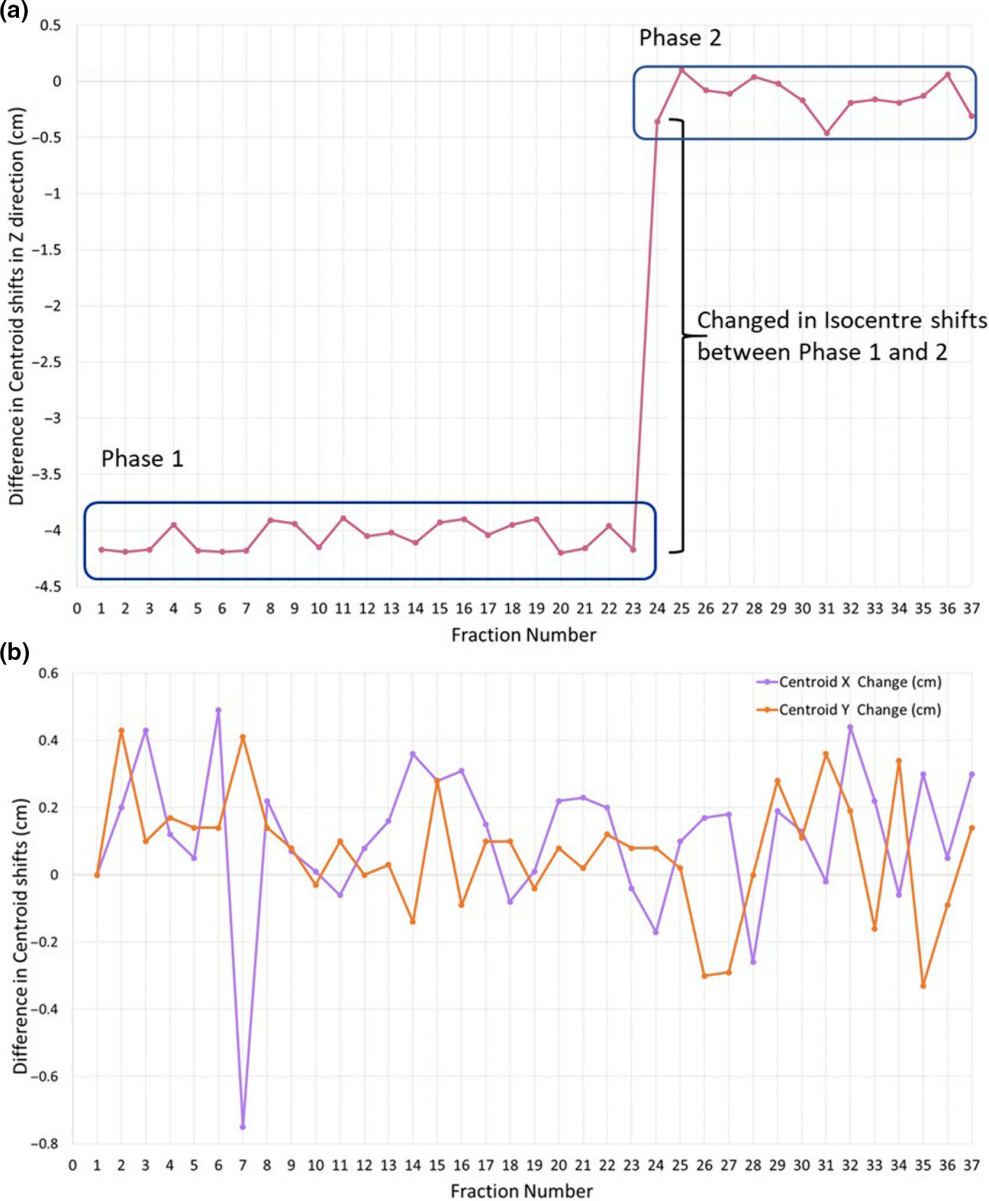
Prostate motion trend analysis shown as the variation in prostate centroid location relative to the CBCT1 baseline. (A) Variation in superior–inferior position; significant change was observed between the 23^rd^ and 24^th^ fraction due to the change in isocentre position. (B) Variation in left–right (purple) and anterior–posterior (orange) position.

**Figure 7:**
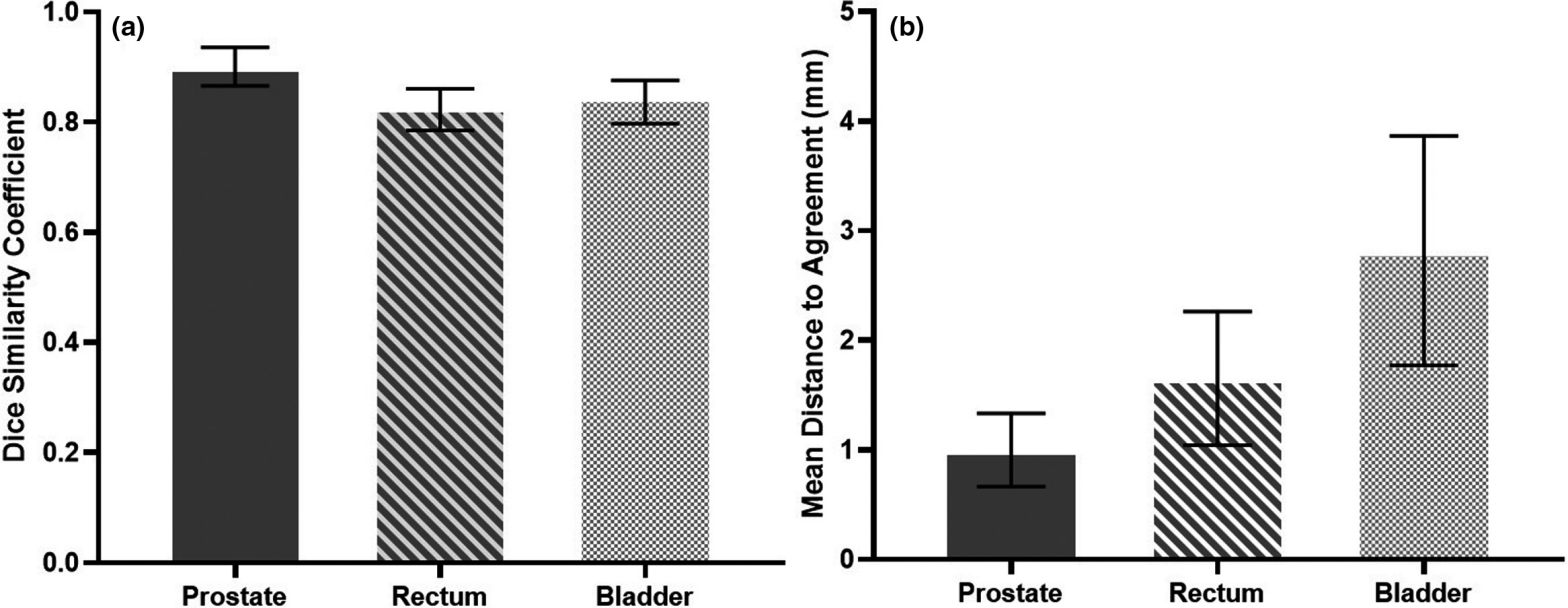
Mean values with error bars representing SD for DSC (A) and MDA (B) of the prostate, rectum and bladder generated based on MIM propagated contours with reference to the RO delineated contours as baseline for 20 patients.

Prostate and OAR contours from pCT were propagated onto the daily CBCTs. The percentage change in prostate and OAR volumes and prostate trend analysis were extracted for analysis, as demonstrated for one example patient in Figures 5 and 6 respectively. Geometrical analysis (mean ± standard deviation (SD)) of the prostate (DSC: 0.9 ± 0.0, MDA: 1.0 ± 0.3 mm), rectum (DSC: 0.8 ± 0.1, MDA: 1.7 ± 0.6 mm) and bladder (DSC: 0.8 ± 0.1, MDA: 2.8 ± 1.0 mm) obtained were within clinically acceptable tolerances (Fig. 7).^7^

## Discussion

This workflow has been successfully commissioned, and we have demonstrated that it is able to accurately perform rigid registration between P1 and P2 plans prior to deforming the P2 doses back to P1 for dose summation. This workflow has proven to successfully accumulate dose and propagate contours with two different isocentres, making it applicable for all HR‐PCa cases that require PLNs irradiation. The geometrical analysis results obtained were comparable to studies using DIR for contour propagation with RO delineated contours as baseline.^8^, ^9^ From the data extracted in this workflow, we have demonstrated it is possible to compare clinically relevant dose parameters between the MIM accumulated dose and the planned dose, and to assess daily volume variation and motion. Similar workflow with single isocentre has been validated previously.^10^

Using accumulated dose estimates to perform dosimetric correlations to patients' toxicity, rather than relying on the initial planned dosimetry, will allow a more accurate reflection of the actual spatial dose received by the patient.^11^ This work could be adapted to other anatomical sites (e.g. thorax and abdominal) with 2‐phased regimens to obtain a more accurate dose estimates of the target and OARs and relating the accumulated dose received to toxicity.

## Conclusion

We have demonstrated that the present workflow using existing commercial systems has shown to be an efficient dose accumulation method to analyse and evaluate the influence of inter‐fractional organs variations on the dose received by the patient when compared to the planned dose on a static CT image. This workflow will now be used to conduct a larger dose accumulation study focusing on correlating the actual dose estimates received by the patient with clinical endpoints for acute and late toxicity.
